# The unexplored relationship between spontaneous osteoclastogenesis and platelets in osteoporosis

**DOI:** 10.3389/fmed.2025.1584022

**Published:** 2025-07-21

**Authors:** Francesca Salamanna, Alberto Di Martino, Deyanira Contartese, Cesare Faldini, Gianluca Giavaresi, Milena Fini

**Affiliations:** ^1^Surgical Sciences and Technologies, IRCCS Istituto Ortopedico Rizzoli, Bologna, Italy; ^2^1st Orthopedic and Traumatologic Department, IRCCS Istituto Ortopedico Rizzoli, Bologna, Italy; ^3^Department of Biomedical and Neuromotor Science-DIBINEM, University of Bologna, Bologna, Italy; ^4^Scientific Direction, IRCCS Istituto Ortopedico Rizzoli, Bologna, Italy

**Keywords:** osteoporosis, spontaneous osteoclastogenesis, platelets, cytokines, chemokines, endothelial cells, immune cells

## Abstract

Spontaneous osteoclastogenesis, a phenomenon characterized by the unregulated differentiation and activation of osteoclasts in the absence of exogenous stimulatory factors, plays a central role in osteoporosis. While conventionally attributed to an imbalance between osteoclast and osteoblast activity, as well as to factors they release and/or produce, and to the involvement of T cells, emerging evidence suggests that platelets may contribute to this process beyond their established role in hemostasis. In this opinion, we propose that platelet activation and the subsequent release of cytokines, growth factors, and chemokines, including PDGF, IL1β, TGFβ, MIP-1α, TNFα, CXCL12 (SDF1), and CCL5 (RANTES), create a pro-inflammatory and osteoclastogenic microenvironment. These mediators may enhance RANKL production, recruit osteoclast precursors, and disrupt osteogenic signaling, indirectly fostering spontaneous osteoclastogenesis. Additionally, platelet interactions with endothelial cells, macrophages, and immune populations could further amplify inflammatory responses and sustain chronic bone resorption, contributing to the stimulation of spontaneous osteoclastogenesis. Although direct evidence linking platelets activation to spontaneous osteoclastogenesis is not yet available, existing literature supports the plausibility of this interplay. Exploring this underrecognized platelet-bone axis could provide new insights into osteoporosis pathophysiology and open avenues for novel diagnostic and therapeutic strategies. These hypotheses may be assessed in clinical practice to develop innovative approaches for the screening, diagnosis, monitoring and treatment of osteoporosis.

## 1 Introduction

Osteoclastogenesis is a physiological process required to support bone remodeling, through which osteoclasts–specialized bone-resorbing cells–are generated and activated from precursor cells in bone marrow (1, 2). This process keeps the dynamic balance between bone formation and resorption, thereby ensuring bone health and structural integrity (2). Differentiation and activation of osteoclasts are primarily regulated by two key signals: receptor activator of nuclear factor κB ligand (RANKL) and macrophage colony-stimulating factor (M-CSF). This latter promotes the proliferation and survival of osteoclast precursors, while RANKL drives their differentiation into mature osteoclasts (3, 4). Under physiological conditions, the balance between bone-forming osteoblasts and bone-resorbing osteoclasts ensures the maintenance of skeletal integrity (5). However, in many chronic, inflammatory and oncologic diseases, such as osteoporosis, rheumatoid arthritis, and multiple myeloma, this balance is disrupted, leading to increased osteoclast activity, accelerated bone loss, and, in severe cases, pathological fractures (6, 7). A particularly significant phenomenon in this pathological context is spontaneous osteoclastogenesis, which differs from the physiological process because it occurs in the absence of direct stimuli (8, 9). In this form of pathological bone resorption, the differentiation and activation of osteoclasts are driven by inflammatory and immunological factors rather than conventional physiological signals (8, 9). Chronic inflammation creates a pro-osteoclastogenic environment characterized by elevated production of inflammatory cytokines, which indirectly stimulate osteoclast formation (8, 9). In rheumatoid arthritis, systemic inflammation and persistent immune cell activation contribute to extensive bone loss (8–10). In Multiple Myeloma, disruption of the regulation of bone formation occurs, promoting spontaneous osteoclastogenesis through interactions between tumor cells and the bone microenvironment, characterized by the inappropriately increased expression of IL6 (8, 9, 11). The result is a pathological state characterized by uncontrolled bone resorption, with severe consequences for skeletal stability and function (8–11). A key example of spontaneous osteoclastogenesis is observed in osteoporosis, a topic that remains relatively underexplored by current research (8, 9, 12, 13). Despite osteoporosis commonly affects a significant portion of the population, the biological implications and underlying mechanisms that lead to the spontaneous activation of osteoclasts in this disease warrant further investigation to better understand the phenomenon and to develop more effective therapeutic strategies (14, 15).

Osteoporosis is characterized by a reduction in bone mineral density (BMD) and increased bone fragility, which significantly elevates the risk of fractures, particularly at vertebrae, femur, and wrist (15). According to the World Health Organization (WHO), osteoporosis approximately affects 200 million people globally (16). In Europe, it is estimated that over 22 million women and around 5 million men suffer from this condition (17). In the United States, about 10% of women over the age of 50 have established osteoporosis, while an additional 44% exhibit signs of osteopenia (18). The prevalence increases with age, with the lifetime risk to develop an osteoporotic fracture estimated at 40% for women and 13% for men above the age of 50 (19). In Italy, recent studies indicate that approximately 4 million women and 1 million men are affected by osteoporosis, with its incidence rising due to the continuous aging of the population (20, 21). Osteoporotic fractures are a significant cause of disability and mortality in older patients, with a substantial impact on healthcare costs (22).

Although the molecular mechanisms underlying osteoporosis are numerous and complex, spontaneous osteoclastogenesis appears to play a pivotal role (12, 13). The decline in estrogen levels not only reduces osteoblast activity but also promotes the activation of T cells, leading to elevated production of pro-inflammatory and pro-osteoclastogenic cytokines, such as TNFα and RANKL, which stimulate “spontaneous” osteoclast activation (12, 13). Therefore, T cells are indispensable for spontaneous osteoclastogenesis in the osteoporotic microenvironment (13, 23, 24). However, considering the common role of T cells in osteoclasts’ formation in several inflammatory bone disorders, it is crucial to identify and understand the specific mechanisms driving this phenomenon in osteoporosis patients (25, 26). At the same time, further research is required to identify additional factors that might modulate or be associated with spontaneous osteoclastogenesis in this pathological context. Recent evidence in this area suggests that platelets and specific hematological parameters - particularly those related to platelet, such as platelet count (PLT), mean platelet volume (MPV), platelet distribution width (PDW), plateletcrit (PCT), platelet-to-lymphocyte ratio (PLR), and red cell distribution width-to-platelet ratio (RPR) - play significant roles in various aspects of the immune system and tissue repair (27). These parameters can release several growth factors and cytokines that may potentially influence osteoclast function and stimulate osteoclastogenesis. Furthermore, in our recent systematic literature review, we highlighted a positive correlation between platelet size, distribution width, volume changes, and low BMD due to osteoporosis (27).

Based on current knowledge, we hypothesize that platelet-derived pro-inflammatory cytokines may indirectly stimulate spontaneous osteoclastogenesis, thereby contributing to the loss of bone mass associated with osteoporosis. Additionally, we postulate that the interaction between platelets and endothelial cells, macrophages, and other immune cells–all key players in bone resorption and pathophysiology of osteoporosis–could mediate this relationship. Although the role of platelets in inflammation and osteoporosis is increasingly recognized, the direct link between platelet parameters and spontaneous osteoclastogenesis remains unexplored. Confirming these hypotheses could significantly enhance osteoporosis screening, diagnosis, monitoring, and treatment strategies, leading to more targeted and timely interventions.

Our hypotheses suggest the potential identification of non-invasive blood biomarkers for clinical use. Moreover, if validated, the modulation of platelet function could emerge as a strategy to counteract spontaneous osteoclastogenesis, reducing the risk of bone fractures and long-term skeletal damage to develop. Understanding how platelets activation influences spontaneous osteoclast formation–or vice versa–could pave the way for novel screening, diagnostic, and therapeutic strategies not only for osteoporosis but also for other pathological conditions characterized by dysfunctional osteoclastogenesis. This perspective may significantly contribute to the understanding of mechanisms of osteoporosis, with the potential to open new scientific avenues and contribute to the development of innovative approaches for the diagnosis and clinical treatment of this condition.

## 2 Spontaneous osteoclastogenesis and osteoporosis

Osteoclastogenesis can be influenced by biological, inflammatory, and hormonal factors (5). From a biological perspective, the Receptor Activator of Nuclear Factor Kappa-B/Receptor Activator of Nuclear Factor Kappa-B Ligand/Osteoprotegerin (RANK/RANKL/OPG) signaling pathway plays a central role (28). RANKL promotes osteoclast formation by binding to RANK present on the surface of osteoclast precursors, while OPG acts as a natural inhibitor of this interaction (3, 4, 28). An increase in RANKL or a reduction in OPG favors osteoclast activation, leading to increased bone resorption.

Several pro-inflammatory cytokines, such as Interleukin 1 Beta (IL1β), Tumor Necrosis Factor Alpha (TNFα), and Interleukin 6 (IL6), can directly stimulate osteoclast formation (28, 29). These molecules are often elevated in pathological conditions such as osteoporosis, in which the inflammatory process contributes to excessive bone resorption (29, 30). Other proteins, such as Transforming Growth Factor Beta (TGFβ) and Platelet-Derived Growth Factor (PDGF), also modulate osteoclast activity in response to inflammatory signals (31).

Hormonal factors also play a crucial role in osteoclastogenesis (30, 32). Estrogen deficiency, typical of post menopause, stimulates osteoclast formation by reducing RANKL inhibition and increasing its expression, ultimately leading to enhanced bone resorption (30, 32). Additionally, parathyroid hormone (PTH), when present at high levels–as in some endocrine disorders–directly stimulates osteoclast formation and accelerates bone resorption (33). Although estrogen deficiency is a recognized driver of osteoclastogenesis, especially in postmenopausal women, men, despite having lower baseline estrogen levels, exhibit a lower incidence of osteoporosis (34). This apparent paradox may be explained by several physiological factors. First, men typically achieve a higher peak bone mass than women, which provides a protective reserve against bone loss (35). Second, the decline in sex hormones in men occurs more gradually with age, as opposed to the abrupt estrogen drop in postmenopausal women (36). Third, testosterone in men can be locally converted into estradiol via aromatase activity within bone tissue, thereby exerting protective effects on bone remodeling (37). Finally, differences in systemic inflammation and immune responses between sexes may further modulate the osteoclastogenic environment (38). Together, these mechanisms likely contribute to the lower susceptibility of men to osteoclastogenesis and osteoporosis, despite their lower circulating estrogen levels. In addition to hormonal and inflammatory factors, vitamin D status also plays a key role in bone homeostasis. Insufficient vitamin D can disrupt the balance between osteoblasts and osteoclasts, favoring osteoclasts activation and bone resorption (39).

The heightened inflammatory and hormonal milieu observed in osteoporotic individuals may also translate into altered cellular behavior. In line with this, several clinical studies have reported the phenomenon of spontaneous osteoclastogenesis in osteoporotic patients compared to healthy controls (12, 13, 27, 40–42). These demonstrated that, even in the absence of stimulators such as M-CSF, TNFα, and RANKL, peripheral blood cultures from osteoporotic patients formed a significantly higher number of osteoclasts when compared with healthy controls (43). However, the addition of M-CSF and RANKL to samples from osteoporotic patients brought the two groups to comparable levels of osteoclastogenesis, counterbalancing the initial differences (40). Cultures from osteoporotic patients also exhibited significantly higher levels of TNFα and RANKL, indicating a greater inflammatory activation (12). Interestingly, supplementation with 1,25-OH vitamin D3 in the cultures reduced osteoclast numbers in both groups while simultaneously enhancing their resorption activity (41). Notably, the lacunar resorption area was significantly larger in osteoporotic patients compared to controls, regardless of vitamin D3 supplementation (41).

A crucial aspect that emerged from other clinical studies is the key role of T cells in the osteoclastogenesis in osteoporotic patients (41). Depleting T cells from cultures drastically reduced osteoclast formation, highlighting their function and role in sustaining cytokine production, such as TNFα and RANKL, mandatory for osteoclastogenesis (41, 42). Furthermore, the use of neutralizing antibodies against TNFα and RANKL significantly reduced spontaneous osteoclast formation, with a particularly pronounced effect in osteoporotic patients (41, 42). These findings suggest that the mechanisms underlying spontaneous osteoclastogenesis and the involvement of inflammatory factors and T cells play a decisive role in the pathogenesis of osteoporosis (41).

## 3 Platelets and platelet-related parameters and osteoporosis

Beyond their classical role in hemostasis, platelets are now increasingly acknowledged for their crucial role in immune and inflammatory processes, which are increasingly implicated in age-related bone loss and osteoporosis (44). These anucleate cells, typically ranging between 150,000 and 400,000/μL, originate from megakaryocytes in the bone marrow and have a lifespan of 7–10 days (45). Despite lacking nuclei, platelets retain significant amounts of messenger RNA (mRNA), microRNAs, and non-coding RNAs (ncRNA), allowing them to regulate mRNA translation, synthesize proteins, and secrete peptides essential for the hemostatic process. Notably, antiplatelet drugs have been shown to modulate immune responses and attenuate platelet-driven inflammation, significantly reducing mortality from infections and sepsis (46, 47).

With aging, however, platelet homeostasis and function undergo significant alterations that may have downstream effects on bone remodeling. In aging individuals, platelets exhibit functional exhaustion characterized by reduced aggregation capacity, spontaneous activation, altered granule secretion, and increased externalization of phosphatidylserine (PS) a hallmark of both apoptosis and activation (48, 49). PS exposure not only enhances platelet procoagulant activity by facilitating thrombin generation but may also amplify local inflammation and immune cell recruitment within the bone microenvironment (48, 49). These changes, combined with the heightened oxidative stress and chronic low-grade inflammation of the aging bone marrow niche, can dysregulate platelet production and function, promoting sustained inflammation and increased osteoclastogenesis (48, 49). This mechanistic link may contribute to bone fragility and the pathogenesis of osteoporosis in the elderly.

Under physiological conditions, platelet reactivity is primarily driven by interactions between their receptors and endothelial cells of damaged vessels. Elevated platelet counts often correlate with megakaryocyte activity and the release of pro-platelet cytokines (50–53). In inflammatory conditions, platelets are recruited to the endothelium through interactions involving selectin and integrin receptors, complement receptors, cytokines, chemokines, and receptors such as ADP, PCY, and PAR-1/4. These interactions lead to platelet activation and integration with the walls of blood vessel, often accompanied by interactions with polymorphonuclear cells (PMNs), monocytes, dendritic cells (DCs), and T and B lymphocytes, triggering local inflammation (54, 55). P-selectin, released by endothelial cells upon injury, also plays a significant role in enhancing platelet activation and in the recruitment of neutrophils and monocytes (56). Activated platelets are characterized by the presence of intracellular adhesion molecules like ICAM-1 and MCP-1, which facilitate neutrophils and monocytes adhesion. Several proinflammatory mediators, including CD154 (CD40 ligand), CXC chemokines (CXCL1, CXCL4, CXCL5, CXCL7, CXCL12), IL8 and TGFβ coordinate leukocyte recruitment and activation. Platelets also release bioactive substances, such as growth factors, cytokines, chemokines, coagulation proteins, proteolytic enzymes, microRNAs, and mRNAs, along with Weibel–Palade bodies and extracellular vesicles, which influence endothelial and immune cells activity (57–59). The inflammatory effects of activated platelets are also associated with the secretion of Platelet-Activating Factor (PAF) and Vascular Endothelial Growth Factor (VEGF), leading to endothelial relaxation and increased leukocyte and platelet recruitment. Additionally, activated platelets stimulate neutrophils within the Neutrophil Extracellular Traps (NET) network and affect the apoptosis of polymorphonuclear neutrophils, influencing cell lifespan and the duration of inflammation (60). The proinflammatory actions of platelets can aggravate the pathogenesis of various diseases, including rheumatoid arthritis and cardiovascular conditions.

From a clinical and diagnostic perspective, several hematological parameters related to platelets are relevant for understanding their role in several biological and pathological processes. For instance, variations in platelet number and related parameters have been associated with increased osteoclastic activity and greater loss of bone mass in osteoporosis. Several clinical studies examined the relationship between platelet and osteoporotic status based on BMD values in several cohorts of women, including healthy, osteopenic, and osteoporotic individuals (61–63). Reduced MPV and PDW levels were found in osteoporotic patients, correlating with BMD T-scores (62). A larger study involving 175 patients (72% osteoporotic) showed that MPV inversely correlates with body mass index (BMI) (62). However, Vural et al. (63), found neither significant differences in MPV and platelet counts, nor a relationship between vitamin D levels and MPV. MPV reflects the average size of circulating platelets and serves as a surrogate marker for platelet activation and turnover (64). Larger platelets are typically younger, more metabolically active, and possess greater pro-inflammatory and procoagulant potential (64). An increased MPV, often seen in states of enhanced thrombopoiesis, may therefore indicate the release of reactive platelets capable of contributing to pathological bone remodeling and promoting osteoclastogenesis in osteoporotic patients (64). D’Amelio et al. (65) reported also that platelet vitamin D receptor expression was lower in osteoporotic patients, correlating with BMD variation and to a worse response to vitamin D, leading to increased PTH levels. Moreover, PLR correlated with low BMD in postmenopausal osteoporotic patients, particularly in the femoral and lumbar regions, also associated with low vitamin D levels (66, 67). Kim et al. (68), investigating the association between peripheral blood cell counts and BMD (evaluated by the DXA T-score), observed that in osteoporotic patients, platelet count correlates with BMD along with white and red blood cell counts. Finally, they found that plasma platelet-activating factor (PAF) levels correlated with vertebral fractures and BMD (69).

Collectively, these findings indicate a significant interplay between platelet function and osteoporosis, highlighting the critical roles of inflammation and immune regulation in this relationship. The interaction between platelets and the bone microenvironment appears to influence osteoclastic activity and bone resorption, suggesting that alterations in platelets behavior may contribute to the pathophysiology of osteoporosis. Further investigation into the mechanistic pathways involved could provide valuable insights into potential therapeutic targets for managing osteoporotic conditions.

## 4 Hypothesized links between spontaneous osteoclastogenesis and platelet related parameters

**FIGURE 1:**
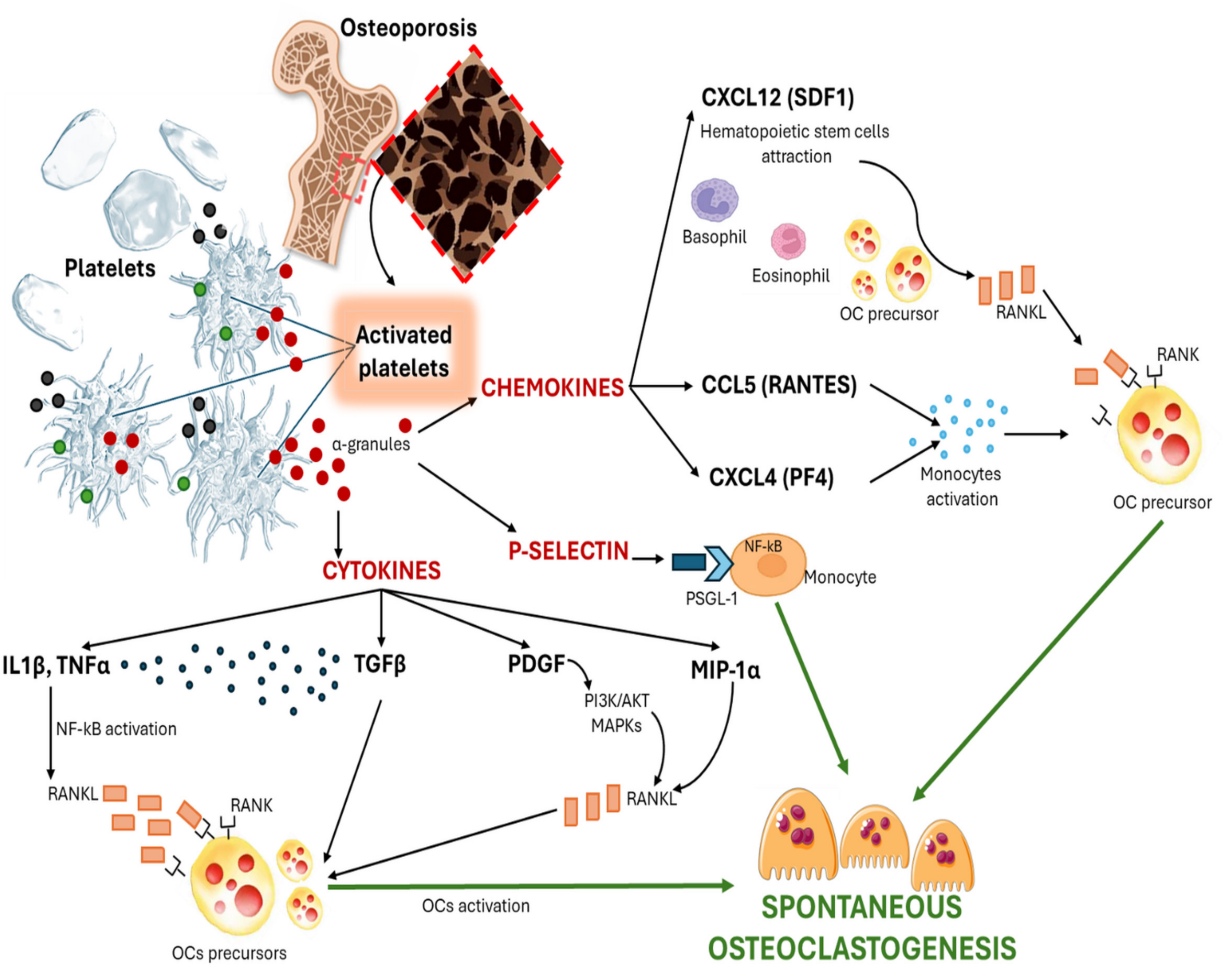
Schematic representation of platelet-mediated release of PDGF, IL1β, TGFβ, MIP-1α, TNFα, CXCL1, CXCL4, CCL5, CXCL12, and P-selectin, contributing to the induction of spontaneous osteoclastogenesis in osteoporosis.

Platelets contain three distinct types of granules: α-granules, dense granules, and lysosomes. Among these, α-granules are the most significant in mediating inflammatory responses. The release of mediators like cytokines, chemokines, and adhesion molecules by platelets enables them to influence various cell types directly and indirectly. This interaction underpins our hypothesis that these mediators may link spontaneous osteoclastogenesis to platelet-related parameters. Specifically, cytokine and chemokine depositions may activate and differentiate osteoclast precursors, while adhesion molecules could affect cell interactions crucial for osteoclasts’ function (Figure 1). We hypothesize that platelet activity significantly impacts osteoclastogenesis, thereby accelerating bone remodeling in osteoporosis and contributing to the loss of bone mass.

### 4.1 Cytokines

Cytokines primarily function to regulate inflammatory processes, particularly by driving the proliferation and differentiation of lymphocytes (70). Platelets secrete several key proinflammatory cytokines, such as platelet-derived growth factor (PDGF), interleukin-1 (IL1), transforming growth factor-beta (TGFβ), macrophage inflammatory protein-1a (MIP-1α), and tumor necrosis factor-alpha (TNFα) (70–72). PDGF exerts its action by binding to specific tyrosine kinase receptors on the membranes of target cells, such as fibroblasts and mesenchymal progenitor cells, thereby activating intracellular signaling pathways, including the PI3K/AKT and MAPK pathways, which are critical for cell survival, migration and proliferation (73, 74). This growth factor also contributes to new bone tissue formation by stimulating angiogenesis, which is essential for the vascularization of regenerating tissue (73, 74). TGFβ operates through the TGFβ receptor complex types I and II, activating intracellular signaling via SMAD proteins (75). In bone tissue, TGFβ exerts a dual effect: it promotes osteoblast proliferation and extracellular matrix formation while physiologically modulating osteoclastogenesis negatively (76); however, under pathological or inflammatory conditions, TGFβ may indirectly promote osteoclastogenesis by enhancing RANKL expression and osteoclast precursor recruitment.

Concerning IL1β and TNFα, these trigger several inflammatory signaling pathways, particularly NF-κB (77). Moreover, these induce the production of RANKL by osteoblasts and bone marrow stromal cells (77, 78). RANKL binds to RANK on the surface of osteoclast precursors, activating intracellular signaling that drives their differentiation, fusion, and activation (77, 78). This process is balanced by the action of OPG, which acts as a decoy receptor for RANKL, preventing its binding to RANK and thereby limiting osteoclast activation (77, 78). In this highly complex and multifaceted microenvironment, we hypothesize that platelet activation and the subsequent release of pro-inflammatory cytokines, including PDGF, IL1β, TGFβ, MIP-1α, and TNFα, critically contribute to the process of spontaneous osteoclastogenesis observed in osteoporosis. Upon activation, platelets release these molecules which influence the bone microenvironment in multiple ways. Although TGFβ physiologically inhibits osteoclastogenesis, the pro-inflammatory environment driven by IL1β and TNFα may transform its role, indirectly amplifying bone resorption (77, 78). Similarly, the overactivation of the PI3K/AKT and MAPK pathways, driven by environmental inflammation induced by PDGF, further stimulates spontaneous osteoclastogenesis by sustaining continuous RANKL production. Moreover, MIP-1α enhances the availability of osteoclasts precursors, thereby further promoting spontaneous osteoclastogenesis (79).

In addition to their effects on spontaneous osteoclastogenesis, several platelet-derived cytokines also participate in the regulation of hematopoiesis within the bone marrow. Notably, certain microenvironmental factors, such as thrombopoietin (TPO), IL6, stem cell factor (SCF), and TGFβ, play dual roles in both megakaryopoiesis and osteoclast differentiation (80–82). TPO, for instance, not only drives megakaryocyte proliferation and maturation but also indirectly enhances osteoclastogenesis through modulation of the RANKL/OPG axis (80, 81). IL6 and TGFβ contribute to both lineages, promoting osteoclast activation under inflammatory conditions while supporting megakaryocyte development (80–83). These observations further support the hypothesis that the bone marrow cytokine milieu, shaped in part by platelet activation, orchestrates interconnected hematopoietic and bone-resorptive processes.

### 4.2 Chemokines and adhesion molecules

Following platelet activation, α-granules release a variety of chemokines, including CXCL1 (β-thromboglobulin), CXCL4 (platelet factor 4, PF4), CCL5 (RANTES, regulated upon activation and normal T cell expressed and secreted), CXCL12 (stromal cell-derived factor-1, SDF1) and adhesion molecules, such as P-selectin (70, 84). Chemokines contribute to the recruitment and differentiation of T lymphocytes and stimulate neutrophil activation as well as macrophage phagocytosis (70). A key molecule secreted by α-granules is P-selectin, a well-known marker of platelet activation. P-selectin also binds with P-selectin glycoprotein ligand-1 (PSGL-1) to mediate platelet adhesion to leukocytes and monocytes, facilitating leukocyte recruitment during inflammatory responses (70, 85, 86). Additionally, P-selectin plays a critical role in the tethering and rolling of leukocytes (86). Following platelet activation, these chemokines may promote spontaneous osteoclastogenesis associated with osteoporosis through the recruitment and differentiation of osteoclasts precursors. In fact, CXCL12 (SDF1) is known to attract hematopoietic progenitor cells, including those destined to become osteoclasts (87). Similarly, CCL5 (RANTES) and CXCL4 (PF4) contribute to the activation of monocytes, which are key osteoclast precursors (88). Simultaneously, factors such as CXCL12 can stimulate the production of RANKL by osteoblasts and stromal cells, directly enhancing spontaneous osteoclast formation (89). Likewise, P-selectin promotes the binding of platelets to leukocytes or monocytes, facilitating cellular recruitment during inflammatory responses (90). This process amplifies local inflammation, a well-known condition that promotes pathological bone resorption. The interaction between P-selectin and its ligand PSGL-1 may further anchor monocytes to bone surfaces, creating optimal conditions for their differentiation into osteoclasts (90).

This concerted release of PDGF, IL1β, TGFβ, MIP-1α, TNFα, CXCL1, CXCL4, CCL5, CXCL12, and P-selectin by platelets establishes a highly pro-inflammatory and pro-osteoclastogenic microenvironment. In most cases, this *milieu* is characterized by excessive RANKL production, enhanced recruitment of osteoclasts precursors, and disruption of osteogenic signaling, ultimately driving spontaneous osteoclastogenesis and leading to uncontrolled bone resorption (Figure 1).

**FIGURE 2:**
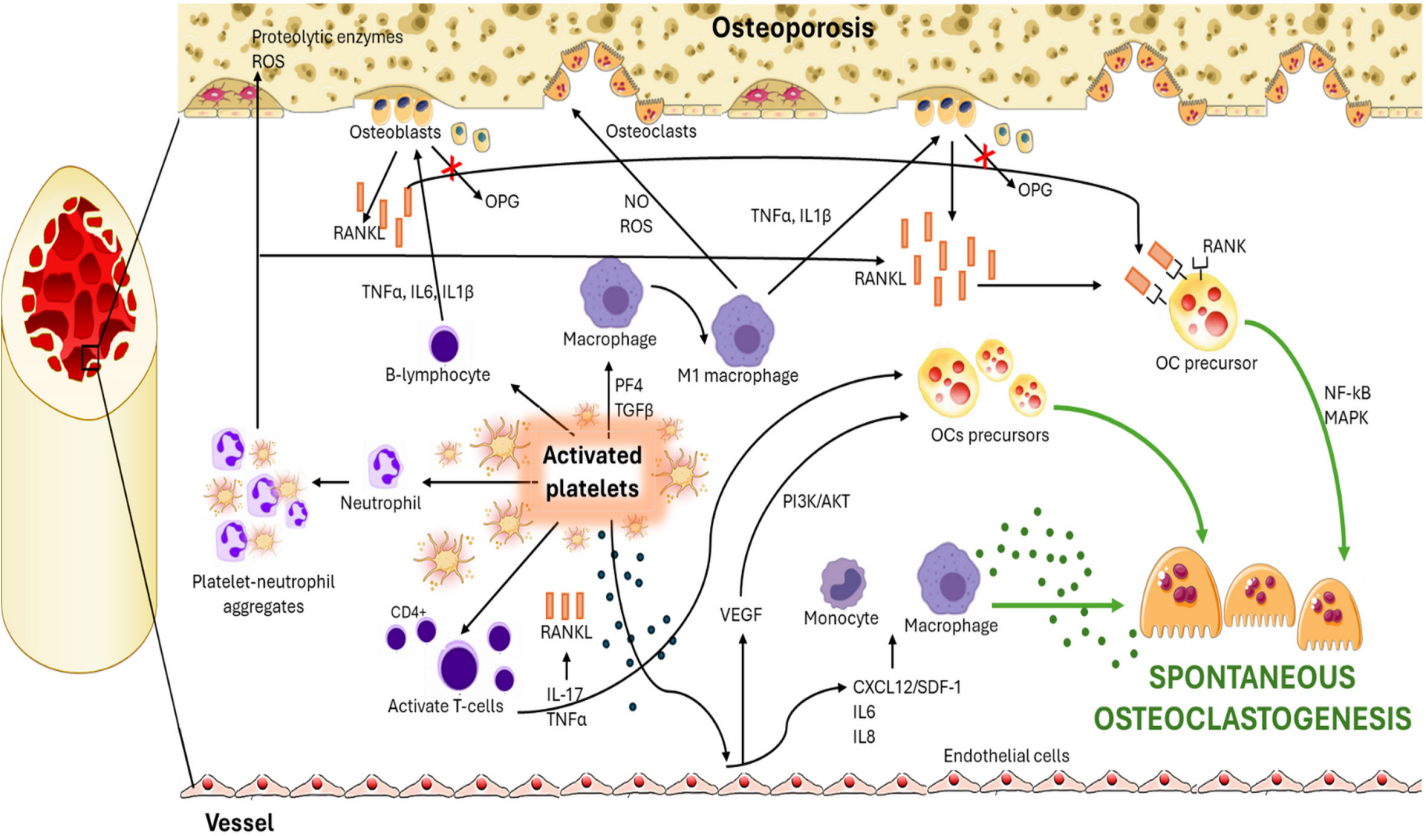
Schematic representation of platelet crosstalk with endothelial cells, macrophages, and lymphocytes, contributing to a persistent inflammatory state that drives the induction of spontaneous osteoclastogenesis in osteoporosis.

A second potential mechanism underlying the crosstalk between platelet and spontaneous osteoclastogenesis in osteoporosis may be mediated by complex interactions between platelets and various key cell types, such as endothelial cells, macrophages, and different immune cell populations, including T lymphocytes, B lymphocytes, and neutrophils (91–94) (Figure 2). These cellular interactions involve intricate molecular dialogues that profoundly influence bone metabolism and the regulation of bone resorption.

### 4.3 Endothelial cells

Endothelial cells, which line the blood vessels within the bone marrow, represent an important interface between the bloodstream and bone tissue (92, 93). In addition to providing structural support for bone vascularization, these cells actively modulate the bone microenvironment. Upon stimulation by activated platelets, endothelial cells can release several soluble factors, including chemokines (e.g., CXCL12/SDF1) and pro-inflammatory cytokines such as IL6 and IL8 (94). These signals facilitate the recruitment and adhesion of macrophages and other immune cells to bone resorption sites. Moreover, endothelial cells can release VEGF, which not only promotes angiogenesis, but also directly influences osteoclast differentiation (95, 96). VEGF stimulates osteoclast precursors through the PI3K/AKT intracellular pathway, enhancing their survival and activation (96, 97). In addition to its well-established role in promoting osteoclast precursor recruitment and activation during inflammation, VEGF also contributes significantly to osteoblast function and bone formation (98). By enhancing angiogenesis within the bone microenvironment, VEGF facilitates the delivery of oxygen, nutrients, and regulatory molecules essential for osteoblast proliferation and differentiation (98). Furthermore, it can directly stimulate osteoblasts through MAPK and PI3K/AKT signaling pathways - pathways also involved in osteoclast regulation, though with distinct functional outcomes depending on the target cell type - promoting matrix production and bone mineralization (98). These observations highlight the dual and context-dependent role of VEGF in bone remodeling, with its effects shifting toward resorption or formation depending on the prevailing inflammatory or reparative conditions within the bone marrow niche.

### 4.4 Macrophages

Activated platelets are also known to release chemical mediators such as Platelet Factor 4 (PF4) and TGFβ, which can modulate macrophage activation and promote their polarization toward a pro-inflammatory (M1) phenotype (99). M1 macrophages release cytokines such as TNFα and IL1β, which potentiate the production of RANKL by osteoblasts and bone marrow stromal cells. RANKL, after its binding to RANK on osteoclast precursors, activates intracellular NF-κB and MAPK pathways to promote their differentiation into mature osteoclasts (100). M1 macrophages can also release nitric oxide (NO) and reactive oxygen species (ROS), contributing to bone matrix damage and amplifying the resorption process (101).

### 4.5 Immune cell populations

Platelets also directly interact with several immune cell populations, such as T lymphocytes, B lymphocytes, and neutrophils (70, 102). Activated platelets can stimulate T lymphocytes to produce cytokines, such as IL-17, which promote spontaneous osteoclastogenesis (103). Notably, CD4 + T lymphocytes can also directly influence osteoclasts precursors by releasing TNFα, further contributing to osteoclasts spontaneous differentiation and bone resorption (104). B lymphocytes also play a role in bone metabolism by producing OPG, a natural antagonist of RANKL (105). However, in the chronic inflammatory environment driven by platelet activation, OPG production may be insufficient to counteract the elevated RANKL levels, thereby promoting osteoclastogenesis and bone resorption (105). Finally, platelets interact with neutrophils to form platelet-neutrophil aggregates, which release proteolytic enzymes and ROS into the bone microenvironment (106). ROS, including superoxide anion (O_2_^–^), hydrogen peroxide (H_2_O_2_), and hydroxyl radical (HO), are generated during aerobic respiration and play dual roles in biological systems. At physiological levels, ROS act as intracellular messengers regulating processes such as cell proliferation, differentiation, apoptosis, and gene expression (107). However, excessive ROS production, often associated with aging, inflammation, or degenerative diseases like osteoporosis, disrupts cellular homeostasis, contributes to spontaneous osteoclastogenesis and to extracellular matrix degradation. These phenomena are confirmed also by previous studies that demonstrated that oxidative stress and excessive production of ROS can disrupt the balance between pro-oxidant and antioxidant defense systems, thereby contributing to bone loss (108).

Collectively, the sustained activation of platelets and their intricate crosstalk with endothelial cells, macrophages, and lymphocytes contribute to a persistent inflammatory state. This chronic disruption destabilizes the delicate balance between bone resorption and formation, ultimately fostering spontaneous osteoclastogenesis and pathological bone loss (Figure 2).

## 5 Discussion

Emerging evidence suggests that the interplay between platelets and spontaneous osteoclastogenesis in the pathogenesis of osteoporosis may have significant clinical implications, particularly as a potential biomarker for disease detection, risk assessment, and therapeutic monitoring. Furthermore, given that osteoporosis is strongly associated with aging, it is also essential to consider how age-related changes in platelet biology contribute to disease pathophysiology. Aging not only alters platelet production and clearance by the spleen and liver but also increases basal inflammatory signaling and thrombotic potential (109, 110). These changes may create a systemic environment that further favors spontaneous osteoclastogenesis, reinforcing the hypothesis that platelet dysfunction in the elderly is a contributing factor to bone loss (48, 111, 112).

Hematological disorders such as myeloproliferative neoplasms (MPNs) provide illustrative examples of how chronic platelet activation and systemic inflammation can coexist with bone loss. Patients with MPNs often exhibit increased platelet counts and enhanced secretory activity, alongside elevated inflammatory cytokine levels. Notably, these individuals also show a higher incidence of osteoporosis, supporting the notion that platelet-driven inflammation plays a mechanistic role in bone resorption (113–115).

The identification of platelet-derived factors involved in spontaneous osteoclastogenesis could enable earlier diagnosis of osteoporosis, even before substantial bone loss or fracture events occur, particularly in asymptomatic individuals. This advancement could pave the way for personalized treatment strategies, distinguishing patients who would benefit most from anti-resorptive therapies, such as bisphosphonates, from those requiring anabolic agents like teriparatide. Moreover, platelet-related biomarkers could serve as dynamic indicators of disease progression and treatment efficacy, offering a less invasive and more responsive alternative to traditional diagnostic tools, such as dual-energy X-ray absorptiometry (DXA) (90). Given the critical role of platelets in both bone remodeling and vascular integrity, these biomarkers might also provide a more comprehensive assessment of fracture risk, surpassing the limitations of BMD evaluation alone. Spontaneous osteoclastogenesis, driven by an inflammatory microenvironment rich in platelet-derived cytokines and chemokines, represents a pathological mechanism that contributes to uncontrolled bone resorption in osteoporosis. Understanding the precise molecular interactions between platelets, osteoclast precursors and inflammatory mediators could not only improve diagnostic accuracy, but also identify new targets for therapeutic intervention. Moreover, the potential crosstalk between bone metabolism and cardiovascular health–mediated by shared platelet-driven pathways–suggests that integrating platelet-based diagnostics could refine the clinical management of osteoporosis, particularly in patients with concurrent cardiovascular comorbidities. If validated, this approach could offer a more accessible, cost-effective alternative to conventional diagnostic methods while also unveiling novel molecular targets for therapeutic intervention. The integration of test panels could further enhance the diagnostic precision, enabling comprehensive profiling of platelet activity and osteoclastogenesis. Dedicated test panels could evaluate specific circulating biomarkers, molecular markers, and biological parameters using methodologies ranging from simple serological tests to advanced flow cytometry for cellular profiling. Integrating these novel panels with existing diagnostic tools–such as DXA, biochemical markers of bone resorption (C-telopeptide, pyridinoline, and deoxypyridinoline) and formation (osteocalcin, procollagen type I propeptide), as well as serum levels of vitamin D, calcium, and phosphorus–could offer a more comprehensive assessment of bone health. This enhanced diagnostic approach would facilitate early detection, refined risk assessment, and improved disease monitoring, ultimately supporting a more personalized osteoporosis management strategy. To refine diagnostic evaluation, different panels could be integrated, analyzing platelet-derived inflammatory biomarkers (e.g., PDGF), key chemokines involved in osteoclast precursor recruitment (e.g., CXCL12/SDF1), platelet activation markers (e.g., P-Selectin/CD62P and platelet-leukocyte aggregates), the RANKL/OPG balance, and endothelial and immune activation factors. Incorporating these parameters into an advanced diagnostic panel could enhance osteoporosis risk stratification, uncover new therapeutic targets, and improve disease management. A multidimensional approach would move osteoporosis diagnosis beyond sole reliance on BMD, adopting a more predictive and personalized model that enables targeted and timely therapeutic interventions.

## 6 Conclusion

This study explores the complex and largely unexplored relationship between spontaneous osteoclastogenesis and platelet function within the context of osteoporosis. By proposing potential mechanistic pathways, we emphasize the emerging role of platelets–not only as critical mediators of hemostasis–but also as influential players in bone remodeling through their interaction with osteoclasts precursors. These interactions could underline the dysregulated bone resorption and spontaneous osteoclastogenesis, which are hallmarks of osteoporosis. We hypothesize that platelet-derived factors, including cytokines and growth factors, serve as crucial mediators in the activation, differentiation, and function of osteoclasts. Furthermore, alterations in platelets count and function may reflect broader disturbances in bone homeostasis, providing valuable insights into the pathophysiology of osteoporosis. The recognition of this novel platelet-osteoclastogenesis axis holds a significant clinical promise. Platelet-related biomarkers could offer new opportunities for early detection, risk stratification, and ongoing monitoring of osteoporosis. These markers might also contribute to the refinement of personalized therapeutic strategies, addressing not only the systemic inflammation, characteristic of osteoporosis, but also its direct impact on bone resorption. However, further research is required to validate these hypotheses, to fully elucidate the molecular pathways involved, and to assess the practical application of platelet-derived parameters in clinical settings. Unraveling the intricate interplay between platelets and spontaneous osteoclastogenesis could open new avenues for both diagnostic and therapeutic innovations, ultimately contributing to the transformation in the management and treatment of osteoporosis and to overall patients’ health.

## Data Availability

The raw data supporting the conclusions of this article will be made available by the authors, without undue reservation.
