# Correction: The unexplored relationship between spontaneous osteoclastogenesis and platelets in osteoporosis

**DOI:** 10.3389/fmed.2025.1670375

**Published:** 2025-08-05

**Authors:** Francesca Salamanna, Alberto Di Martino, Deyanira Contartese, Cesare Faldini, Gianluca Giavaresi, Milena Fini

**Affiliations:** ^1^Surgical Sciences and Technologies, IRCCS Istituto Ortopedico Rizzoli, Bologna, Italy; ^2^1st Orthopedic and Traumatologic Department, IRCCS Istituto Ortopedico Rizzoli, Bologna, Italy; ^3^Department of Biomedical and Neuromotor Science-DIBINEM, University of Bologna, Bologna, Italy; ^4^Scientific Direction, IRCCS Istituto Ortopedico Rizzoli, Bologna, Italy

**Keywords:** osteoporosis, spontaneous osteoclastogenesis, platelets, cytokines, chemokines, endothelial cells, immune cells

An incorrect Funding statement was provided. The correct funding statement reads:

The author(s) declare that financial support was received for the research and/or publication of this article. This study was funded by “Finanziato dall'Unione europea—Next Generation EU—PNRR M6C2 - Investimento 2.1 Valorizzazione e potenziamento della ricerca biomedica del SSN - PNRR-POC-2023-12378461”—“Sustainable Innovation with a Method Based on Peripheral Mononuclear Cells to Screen, Monitor and Stratify the Population at Risk of Osteoporosis and Fractures – Discern”.

The original version of this article has been updated.

